# Nonlinear Ultrasonic Time-Domain Identification Based on Chaos Sensitivity and Its Application to Fatigue Detection of U71Mn Rail Steels

**DOI:** 10.3390/s26072262

**Published:** 2026-04-06

**Authors:** Hongzhao Li, Mengfei Cheng, Chengzhong Luo, Weiwei Zhang, Jing Wu, Hongwei Ma

**Affiliations:** 1 School of Mechanics and Construction Engineering, Jinan University, Guangzhou 510632, China; 2 School of Environment and Civil Engineering, Dongguan University of Technology, Dongguan 523808, China; 3 Institute of New Energy, Dongguan 523830, China; 4 Guangdong Provincial Key Laboratory of Intelligent Disaster Prevention and Emergency Technologies for Urban Lifeline Engineering, Dongguan 523808, China

**Keywords:** nonlinear ultrasonic technology, chaos systems, second harmonic, fatigue crack, U71Mn steel

## Abstract

**Highlights:**

**What are the main findings?**

- The proposed method can directly extract the amplitude of nonlinear signals in the time domain.
- It achieves higher identification accuracy than existing frequency-domain methods.

**What are the implications of the main findings?**

- Dual-peak characteristics in $\beta^{\mathrm{amp}}$ are observed during fatigue progression of U71Mn steel.
- The method effectively evaluates early fatigue damage for U71Mn steel.

**Abstract:**

A nonlinear ultrasonic time-domain identification method based on chaos sensitivity was proposed in this study. The Duffing chaotic system was introduced into the weak second harmonic identification to realize early detection and quantitative evaluation of fatigue damage in U71Mn steel. First, to ensure the reliability of nonlinear ultrasonic testing, a probe-pressure monitoring device was designed. Through pressure-stability experiments, 16 N was determined as the optimal pressure, which effectively suppresses contact nonlinearity interference and ensures coupling stability. Subsequently, the Duffing chaos detection system was established. The signal-system frequency-matching problem was resolved through time-scale transformation. Simultaneously, the issue of unknown initial phases was resolved using phase traversal compensation. Based on the chaotic system’s sensitivity to specific frequency signals and immunity to noise, the amplitudes of the fundamental wave and second harmonics in the target signals were quantified to calculate the nonlinear coefficient. Experimental results demonstrate that the proposed method can extract these amplitudes directly in the time domain, thereby effectively overcoming the spectral leakage inherent in traditional frequency-domain methods. The nonlinear coefficient of U71Mn steel exhibits a “double-peak” characteristic as fatigue damage increases. Specifically, the first peak appears at approximately 50% of fatigue life, while the second occurs at approximately 80%. This phenomenon is closely correlated with the distinct stages of internal fatigue crack propagation, reflecting a complex damage-evolution mechanism. This study not only provides a novel method for the precise extraction of weak nonlinear signals but also establishes a critical theoretical and experimental foundation for accurate fatigue life prediction for U71Mn rail steel.

## 1. Introduction

As a core component of the railway transport system, rails are subjected to long-term vehicle loads and complex environmental conditions. The risks of sudden rail fractures due to fatigue and the associated economic losses have become increasingly prominent [1,2]. The safety and reliability of rails directly impact the efficiency and security of railway transportation. Therefore, nondestructive testing of rails has long been studied as a critical topic in the field of railway transport. Ultrasonic nondestructive testing technology has become the primary method for rail inspection due to its safety, efficiency, and cost-effectiveness [3]. However, most existing ultrasonic testing methods are based on the linear ultrasonic theory, which can effectively detect only macroscopic defects but not microscopic defects [4]. This limitation poses a substantial potential threat to the safe operation of railway tracks. U71Mn steel is extensively utilized in railways due to its superior strength and wear resistance. Consequently, conducting an in-depth investigation into the nonlinear ultrasonic response of U71Mn rail steel during fatigue is of significant engineering value for achieving the accurate assessment of early-stage rail damage.

The evolution of fatigue damage in steel is generally divided into four stages: micro-defect initiation, fatigue crack formation and slow propagation, rapid propagation of macroscopic cracks, and final unstable fracture [5]. The emergence of macroscopic cracks indicates that the rail has entered the end stage of its service life. During this stage, the crack propagation rate is exceptionally high, leaving a critically narrow window for effective maintenance and posing a significant risk of sudden rail fracture. Existing studies have shown that the second-order and third-order elastic constants of materials change significantly during the early fatigue damage stage [6]. This change modulates the propagating elastic waves, resulting in a nonlinear effect in the response signal. By analyzing this nonlinear acoustic response, identification and evaluation of early fatigue damage in materials are possible [7,8]. The nonlinearity coefficient [9], typically defined as the ratio of the second harmonic amplitude to the square of the fundamental wave amplitude, serves as a key indicator in nonlinear ultrasonic testing. By quantitatively relating this parameter to fatigue damage variables (e.g., number of cycles, crack length), the material damage state can be accurately assessed.

However, direct observation in the time domain is challenging, as the second harmonic is typically a weak signal that is not only susceptible to noise interference but also obscured by the fundamental wave. Consequently, researchers predominantly rely on frequency-domain analysis, wherein they transform the test signal to extract the amplitudes of the fundamental wave and second harmonics to calculate the nonlinear coefficients [10]. However, this approach introduces a contradiction: while the theoretical models of nonlinear ultrasonics are grounded in time-domain physical mechanisms, the prevailing methods for determining the nonlinearity coefficient rely almost exclusively on frequency-domain analysis. This discrepancy leads to two critical issues. First, the connection between the analytical results and underlying physical mechanisms becomes indirect, hindering the in-depth exploration of intrinsic principles. Second, frequency-domain methods lack robustness in high-noise environments, impeding accurate capture of the weak nonlinear responses induced by early-stage damage and leading to large errors. Recently, a chaotic sensitivity-based weak signal detection method [11] has offered a promising time-domain alternative.

Intrinsic sensitivity is a core dynamical feature of chaotic systems. This characteristic manifests as extreme sensitivity to minute variations in initial conditions and system parameters while exhibiting strong immunity to noise. For instance, Shi et al. [12] constructed a detection system based on the Duffing oscillator to detect the target signals’ frequency. Their simulation study demonstrated that the resonant frequency could be stably captured even in environments with a signal-to-noise ratio as low as −123 dB, thereby validating the high accuracy and stability of the chaotic system in detecting weak sinusoidal resonant signals. Yan et al. [13] established a detection system based on a high-order Duffing oscillator. They applied the system variable zero-crossing equidistance method to record the critical times at which the system transitions into and out of the large-scale periodic state. The phase difference calculated from these timestamps was then utilized to identify phase information and achieve signal demodulation. Wang et al. [14] effectively identified the amplitude of sinusoidal signals by analyzing the periodicity of intermittent chaos and employing an array of chaotic detection systems to cover the target frequency range. Furthermore, they achieved phase identification by measuring the frequency difference associated with the periodic state transitions of intermittent chaos. Additionally, in Ref. [15], the phase of the driving force was introduced through time-delay modulation. This approach exploits the variation in Floquet exponents with time delay, and after empirical correction, the approach successfully identifies the phase. Collectively, the aforementioned studies demonstrate the capability to identify sine and cosine signals embedded in Gaussian white noise and verify the potential of the Duffing equations in the identification of frequency, amplitude, and phase of weak signals.

In 2012, Zhang et al. [16,17,18] pioneered the application of the Duffing chaotic system to guided wave detection. Their work systematically addressed the matching problem between system parameters and modulated wave signals. Consequently, they established a highly sensitive method for identifying weak modulated wave parameters, which enabled the detection of weak ultrasonic guided wave signals generated by small defects, even in strong noise backgrounds. Liu [19] utilized a chaotic system to evaluate the relative strength of the second harmonic generated by weak contact and employed the Lyapunov exponent to detect delamination damage in composite plates. This method demonstrated superior noise immunity and improved the efficiency of second harmonic identification. Hu et al. [20] utilized the Duffing–Holmes system to process nonlinear guided wave signals, qualitatively evaluating the relative harmonic amplitude by Lyapunov exponents. They established a linear relationship between the Lyapunov exponent and crack size, thus successfully enabling the detection of microcracks. Liu et al. [21,22] utilized a Duffing–Van der Pol oscillator for second harmonic detection, achieving quantitative amplitude identification by the periodic trajectory area index (PTAE). However, the above-mentioned methods established only a qualitative relationship between the chaos-based indicators and signal amplitudes or relied on indirect indices that could not directly quantify the signal amplitude in the time domain. Recently, Cheng et al. [23] proposed a quantitative identification method for weak signals using double Duffing oscillators. Their study first analyzed how the phase difference, introduced by signal interception, affected modulated wave amplitude identification. To address this, they applied phase compensation to the driving force. This step established a quantitative relationship between the driving force amplitude and the guided wave amplitude, thereby achieving high-precision identification. The study provided a significant theoretical basis for addressing errors caused by phase differences in harmonic identification.

The present study used the chaotic detection theory to accurately measure the ultrasonic nonlinearity coefficient and applied it to the evaluation of fatigue damage in U71Mn steel. In contrast to conventional methods that rely on frequency-domain amplitude characteristics, the proposed approach extracted the amplitudes of the fundamental wave and second harmonics directly from time-domain signals. This method effectively overcomes inherent limitations in traditional techniques, such as ambiguous physical interpretation, susceptibility to noise, and errors induced by spectral leakage [24,25]. Consequently, the identification accuracies of both the fundamental wave and the second harmonic were significantly enhanced. Building upon this methodology, the fatigue damage characteristics of U71Mn steel were investigated, and a quantitative relationship between the nonlinearity coefficient and the degree of fatigue damage was established. Ultimately, this work provided a critical foundation for the early nondestructive testing of U71Mn steel rails.

The structure of this paper is as follows. Section 2 details the experimental setup, which includes a designed pressure monitoring device that applies controlled static pressure to the probe, thus enhancing the reliability of nonlinear ultrasonic testing. Section 3 describes a chaotic system to extract the amplitudes of the fundamental wave and harmonic waves to calculate the nonlinearity coefficient. Section 4 analyzes the correlation between the nonlinearity coefficient and the degree of fatigue damage in U71Mn steel. Furthermore, the results are compared with existing literature to elucidate the fundamental characteristics of fatigue damage evolution. Finally, Section 5 presents the conclusions and summarizes the key findings of this study.

## 2. Materials and Methods

**Figure 1:**
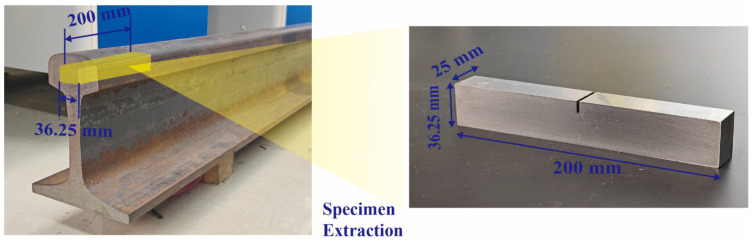
Extraction of specimens from rails.

Specimens were extracted from an undamaged rail conforming to the Chinese standard for 60 kg/m rails, as illustrated in Figure 1. In accordance with the Chinese standard GB 4161-1984 for fracture toughness testing [26], each specimen was machined to the dimensions of 200 mm× 36.25 mm× 25 mm (length × width × height). To facilitate crack initiation, a notch 25 mm in length, 0.2 mm in width, and 10 mm in depth was fabricated at the center of each specimen by wire electrical discharge machining (WEDM). Subsequently, to eliminate the recast layer and localized thermal stresses induced by the WEDM process, the notch roots and specimen surfaces were subjected to progressive grinding using silicon carbide abrasive papers with grit sizes ranging from 400 to 2000. A total of 15 specimens were prepared using identical procedures. Among these, five were utilized to determine the fatigue life of the material, nine were subjected to varying degrees of fatigue loading, and one intact specimen was retained as a reference. Following the fatigue treatment, nonlinear ultrasonic testing was conducted to investigate the correlation between the degree of fatigue damage and nonlinearity coefficient.

### 2.1. Preparation of Specimens with Fatigue Cracks

**Figure 2:**
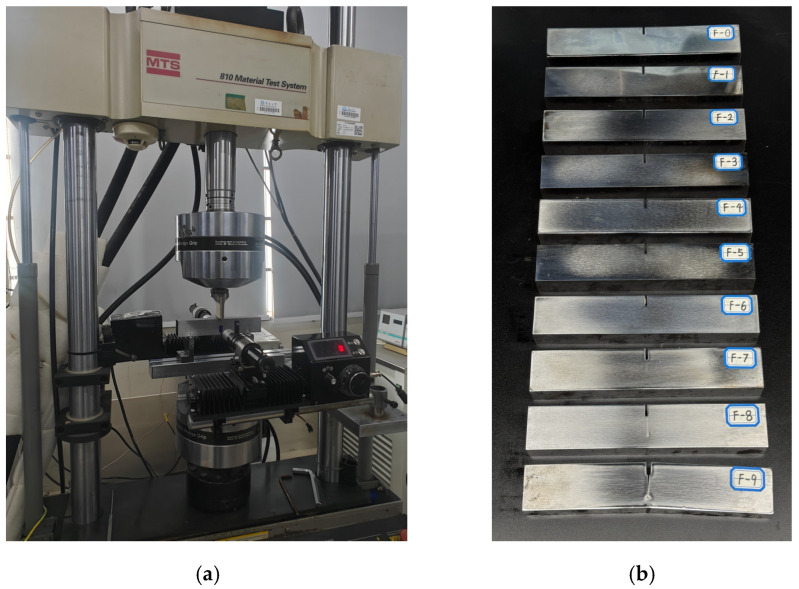
Preparation of specimens with fatigue cracks: (**a**) prefabrication and (**b**) specimens with fatigue cracks.

Fatigue pre-cracking was conducted using an MTS 810 (MTS Systems Corp., Eden Prairie, MN, USA) fatigue testing system. As illustrated in Figure 2a, cyclic loading was applied in a three-point bending configuration to ensure that cracks would initiate, propagate, and fracture from the stress concentration zone at the tip of the wire-cut notch. The loading parameters were set as follows: frequency of 10 Hz, maximum load of 18 kN, and stress ratio of 0.1. The total fatigue life of the specimens was first determined using a crack opening angle of 5° as a failure criterion. Preliminary experiments on five specimens yielded an average fatigue life of N = 138,600 cycles. Based on this reference life, a loading interval of ΔN=15,400 was adopted. By precisely controlling the number of loading cycles, a series of specimens with varying degrees of cumulative damage were prepared. Each specimen was labeled as “F-a”, where “F“ denotes fatigue and “a“ represents the multiple of ΔN. The degree of fatigue damage is defined in Equation (1):

(1) $$Fd_{a}=\frac{a\Delta N}{N}\times 100\%$$

The experimental cases are listed in Table 1, and the specimens subjected to fatigue damage are illustrated in Figure 2b. Specimen F-0 was not subjected to fatigue loading and served as the undamaged reference block. These specimens were subsequently utilized in nonlinear ultrasonic testing to establish the quantitative relationship between the nonlinearity parameter and the degree of fatigue damage.

### 2.2. Nonlinear Ultrasonic Detection System Construction and Probe Coupling Stability Control

**Figure 3:**
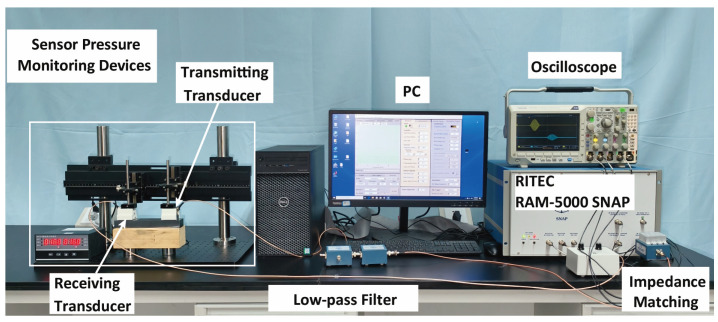
Experimental setup.

Nonlinear ultrasonic measurements were performed on the fatigued specimens using a RITEC RAM-5000-SNAP system (RITEC Inc., Warwick, RI, USA), and the experimental setup is illustrated in Figure 3. By comprehensively considering the detection resolution and acoustic attenuation effect, an oblique probe with a center frequency of 0.5 MHz and 63° was selected as the transmitter, whereas a 1 MHz, 63° probe was employed as the receiver. They were positioned symmetrically relative to the notch, with a separation distance of 100 mm. The high-power output of the SNAP system was connected to a 50 Ω impedance-matching circuit and 0.5 MHz low-pass filter to suppress intrinsic system harmonics. The receiving probe was connected directly to the receiver channel. The excitation signal adopted a Hanning-windowed sinusoidal tone burst, which is expressed as Equation (2):

(2) $$s(t)=A\left(1-\cos\frac{2\pi ft}{n}\right)\sin\left(2\pi ft\right)$$

where A denotes the amplitude, n denotes the number of cycles, and f denotes the center frequency. For this study, n=20 and f=0.5 MHz were selected. This configuration provides an optimal balance between spectral purity—critical for identifying weak nonlinear harmonics—and temporal resolution, ensuring that the target wave packets remain distinguishable from potential interference such as boundary reflections.

**Figure 4:**
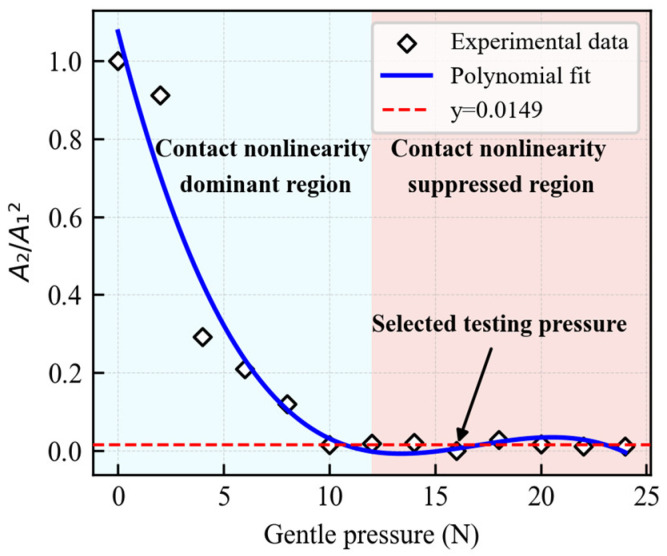
Variation in β under different pressures.

Prior studies [27] have indicated that the coupling conditions between the probe and the specimen can significantly influence nonlinear ultrasonic measurements. To minimize the impact of coupling variations on the nonlinearity coefficient, a designed fixture equipped with a pressure sensor was employed to apply controlled static pressure to the probe, enabling real-time monitoring and feedback. To determine the optimal pressure, a stability test was performed to evaluate the consistency of the nonlinearity coefficient under varying pressure levels. The pressure was incrementally increased from 0 to 24 N in steps of 2 N using an intact specimen secured by the fixture. At each pressure level, five measurements were recorded. The received signals were processed using the Fast Fourier Transform (FFT) to extract the fundamental wave and second harmonic amplitudes in the frequency domain. The variations in the normalized nonlinearity coefficient β are presented in Figure 4. The results indicate that when the pressure exceeds 12 N, the fluctuation in β diminishes significantly, suggesting that the coupling condition has stabilized. To ensure a robust safety margin and operate well within this stable plateau, a static pressure of 16 N was selected as the optimal preload. All subsequent nonlinear ultrasonic experiments maintained this preload to ensure measurement consistency.

**Figure 5:**
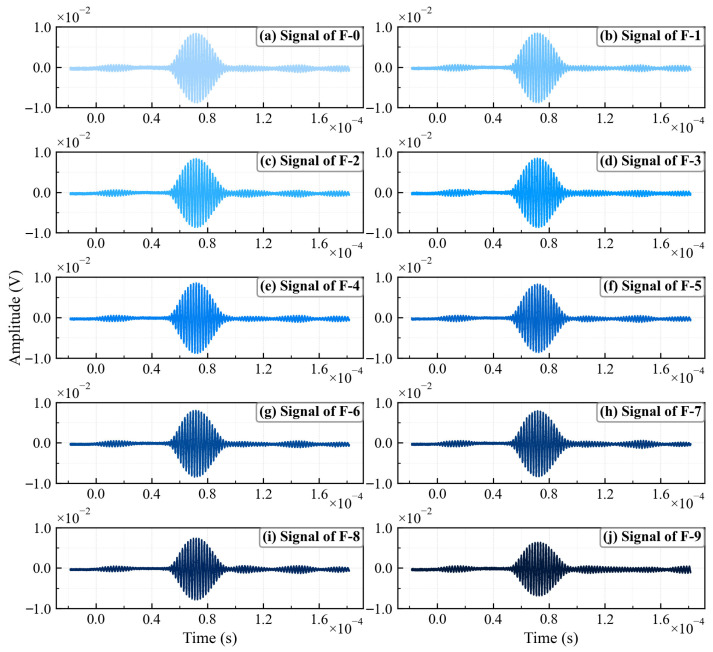
Time-domain signals for F-0 through F-9.

Specimens F-0 through F-9 were tested sequentially. Signal acquisition was performed using a digital oscilloscope with a sampling rate of 100 MHz and a time window of 200 μs. Each recorded waveform was averaged over 64 acquisitions. The results are shown in Figure 5. No significant difference is observed in the time domain of working conditions F-0 to F-8. To ensure the robustness and repeatability of the results, five independent experiments were conducted for each test condition. The results indicate that signal variations induced by fatigue cracks are extremely weak in the time domain, which leads to the conclusion that it is difficult for conventional analytical methods to effectively determine the degree of damage. Therefore, there is an urgent need to develop a new method applicable to the detection of such weak signals. Different from traditional frequency-domain nonlinear analysis, this paper proposes a nonlinear ultrasonic time-domain identification method based on chaos sensitivity. The proposed approach directly analyzes and processes the time-domain signal. The method directly analyzes the time-domain signal to extract the second harmonic amplitude induced by fatigue cracks.

## 3. Data Processing

### 3.1. Nonlinear Harmonics Induced by Fatigue Cracks

**Figure 6:**
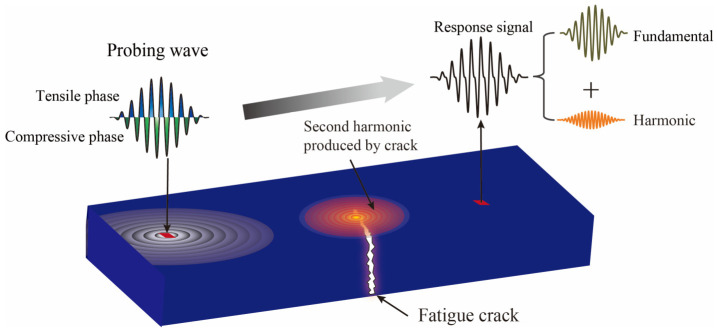
Fatigue crack detection.

Ultrasonic waves propagating through a fatigue crack induce a “breathing” behavior in the defect. As illustrated in Figure 6, when interacting with the incident wave, the crack interface functions as a secondary wave source.

During the compressive phase of the wave, the crack faces close, allowing for the effective transmission of compressive stress. Conversely, during the tensile phase, the crack opens, preventing the transmission of tensile stress across the interface. This asymmetry results in a “force deficit” [28], consequently generating nonlinear effects. Let $F_{\mathrm{eq}}$ denote this equivalent force at the crack interface [29], which is expressed as Equation (3):

(3) $$\begin{array}{l} F_{\mathrm{eq}}=\int_{\Sigma }-\sigma \cdot \vec{x}ds\cdot s(t)\cdot H\\ H=\left\{\begin{array}{l} 1\;\mathrm{Crack}\;\mathrm{open}\\ 0\;\mathrm{Crack}\;\mathrm{closed} \end{array}\right. \end{array}$$

where ∑ denotes the crack opening area under the excitation of the probing wave s(t), and σ represents the stress field induced on the crack surface. $\vec{x}$ is the unit vector along the propagation direction of the guided wave. H is the step function, which describes the characteristics of the periodic opening and closing of the crack. As indicated by Equation (3), $F_{\mathrm{eq}}$ exhibits a discontinuity in the time domain. This discontinuity causes the equivalent force to exhibit nonlinear characteristics, generating higher-order harmonics in addition to the fundamental wave. In particular, the equivalent force $F_{\mathrm{eq}}^{2\omega }$ that generates the second harmonic is expressed as Equation (4):

(4) $$F_{\mathrm{eq}}^{2\omega }=A_{2\omega }\cdot \left|F_{\mathrm{eq}}\right|$$

where $A_{2\omega }$ denotes the amplitude of the equalizing force that generates the second harmonic (2ω). The nonlinear interaction between the crack and ultrasonic waves is represented as an equivalent concentrated force acting at the crack site. This force excites a harmonic displacement field at the free surface [30]. The far field excited by this equivalent force can be expressed as Equation (5):

(5) $$u^{2\omega }=F_{\mathrm{eq}}^{2\omega }\cdot V_{R}(x_{3})H_{1}^{2}(k_{R2}r)\sin(2\omega t-\varphi )$$

where $V_{R}(x_{3})$ denotes the modal displacement function of the ultrasonic wave across the thickness direction, $k_{R2}$ is the wavenumber at the frequency of 2ω, and $H_{1}^{2}$ represents the second-kind Hankel function. The parameter r defines the distance from the equivalent crack source to the sensor, and φ signifies the phase delay of the second harmonic relative to the excitation force. This formulation is employed to characterize the second harmonic induced by fatigue crack nonlinearity, thereby bridging the gap between the theoretical model and the time-domain extraction results.

As indicated by Equations (2) and (3), the amplitude of the equivalent force is closely correlated with the crack opening area under the excitation of the probing wave. A clear functional relationship exists between the amplitude of the second harmonic displacement field and the crack area, providing a theoretical basis for the quantitative detection of fatigue cracks. By accurately measuring the second harmonic displacement field, a quantitative evaluation of the degree of fatigue damage can be achieved. Finally, to eliminate the influence of fluctuations in the excitation signal amplitude, a nonlinear index is adopted to evaluate the fatigue damage degree, which is defined as Equation (6):

(6) $$\beta^{\mathrm{amp}}=\frac{u^{2\omega }}{{\left(u^{\omega }\right)}^{2}}$$

Here, $u^{\omega }$ denotes the displacement induced by the probe wave s(t), and $u^{2\omega }$ represents the displacement arising from the second harmonic generated by the crack. The coefficient $\beta^{\mathrm{amp}}$ quantifies the relationship between the acoustic nonlinear response and crack area, thereby facilitating the quantitative assessment of fatigue damage degree through experimental measurements. Notably, the second harmonic displacement field $u^{2\omega }$ described in Equation (6) corresponds to the time-domain amplitude of the second harmonic in the actual measurement. To distinguish it from the traditional nonlinear coefficient β derived from frequency-domain amplitudes, the superscript “amp” is introduced to denote the time-domain formulation.

However, existing studies predominantly rely on frequency-domain analysis methods, such as the FFT, to determine β. This approach is subject to inherent limitations: First, spectral leakage and windowing effects during the frequency-domain transformation introduce intrinsic errors, leading to distortion in amplitude measurements. Second, weak second harmonic signals are highly susceptible to being submerged in noise, causing traditional frequency-domain methods to suffer from insufficient extraction accuracy. Consequently, the reliability of quantitative evaluations is constrained. To address this challenge, this study proposed a time-domain signal processing method based on chaos theory. This method circumvented the need for frequency-domain conversion, enabling the high-precision extraction of second harmonic amplitudes directly from raw time-domain signals, even within high-noise environments. A detailed discussion of this method is presented in the following section.

### 3.2. Time-Domain Signal Amplitude Identification Based on Chaos Sensitivity

**Figure 7:**
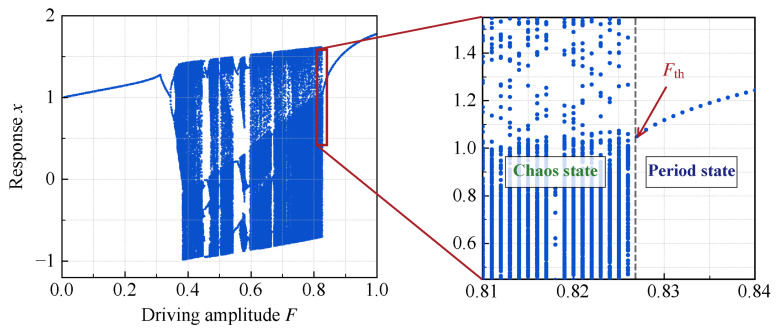
Bifurcation of the Duffing equation.

As a typical chaotic system, the Duffing equation is extensively used to detect weak signals [31]. In this study, the Duffing equation presented in Equation (7) is selected as the signal amplitude identification system, with its expression given by

(7) $$\ddot{x}+k\dot{x}-x+x^{3}=F\sin t$$

where x denotes the system response, k represents the damping coefficient, and Fsint constitutes the external driving term, with F representing the driving amplitude. As indicated by Equation (7), the angular frequency of the driving term is fixed at 1. When the damping coefficient k is fixed, adjusting the driving amplitude F can drive the system into a critical state, marking the transition from a chaotic state to a periodic state. In this state, if a weak signal with the same angular frequency as the driving is inputted as a perturbation term, the system undergoes a state transition, thereby enabling the detection of the weak signal. Therefore, the precise determination of the critical driving amplitude at which the system transition occurs is crucial to this detection method. Based on prior research [32], the damping coefficient was set to k=0.5. Subsequently, the bifurcation of the system response x with respect to the driving amplitude F was obtained through numerical simulation, with F ranging from 0 to 1 (as illustrated in Figure 7). The analysis of the bifurcation characteristics demonstrates that at F= 0.826808, the system transitions precisely from a chaotic state to a periodic state. Consequently, $F_{\mathrm{th}}=\;0.826808$ is defined as the critical driving amplitude for the system state transition.

It should be pointed out that the Duffing equation is only sensitive to signals with the same frequency as their driving force [14]. Therefore, it is necessary to match the target signal to the driving frequency of the detection system. Existing research typically adjusts the driving frequency of the Duffing equation to match the target signal frequency [23]. However, nonlinear ultrasonic signals simultaneously contain fundamental and second harmonic, and a chaotic detection system cannot simultaneously identify signals at two different frequencies. To address this problem, this paper proposes a signal–system frequency matching method based on time-scale transformation, and the details are as follows. First, the detection system uses the Duffing equation shown in Equation (7), with the driving circle frequency fixed at 1. Then, perform a time-scale transformation on the target signal: for the signal shown in Equation (7), let $t=\frac{1}{2\pi f}\tau$, where 2πf is the time-shift coefficient, t represents the original time, τ denotes the transformed time, and f is the frequency of the target signal. This time scale transformation essentially stretches and compresses time, thereby changing the period and frequency of the target signal to match the detection system. Substituting the above time scale transformation into Equation (2) yields:

(8) $$s(\tau )=A\left(1-\cos\frac{\tau }{n}\right)\sin\left(\tau \right)$$

It can be seen from Equation (8) that the signal circle frequency after time-scale transformation is 1, which is the same as the driving frequency of the detection system. Obviously, the frequencies of the fundamental and second harmonics can be shifted to 1 by time scale transformation, thereby matching the system. This enables the use of a chaos detection system to simultaneously identify the fundamental frequency and the second harmonic.

**Figure 8:**
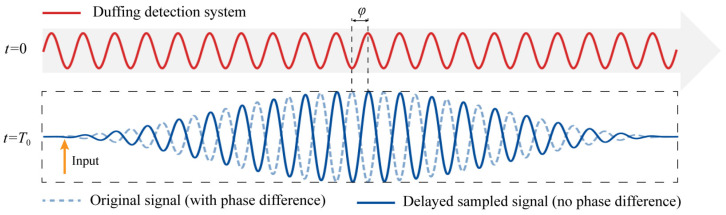
Delay sampling of signals to eliminate phase differences.

For notational convenience, the transformed time τ is redefined as t. Superimposing Equation (8) onto the driving term of the Duffing equation maintained at a critical chaotic state yields Equation (9):

(9) $$\ddot{x}+0.5\dot{x}-x+x^{3}=F_{\mathrm{th}}\sin\left(t+\varphi \right)+A\left(1-\cos\frac{t}{n}\right)\sin\left(t\right)$$

where φ denotes the phase difference between the driving term of the Duffing equation and the target signal. In the case of the second harmonic, both detection distance and damage induce a phase shift in the second harmonic wave relative to the fundamental wave. As the signal acquisition is time-referenced to the fundamental wave, an initial phase difference arises between the second harmonic and the driving term. Specifically, the excitation term of the Duffing equation starts running at *t* = 0. However, the second harmonic is input into the equation at $t=T_{0}$. Therefore, when the second harmonic is input, the excitation term generates an initial phase φ. This results in a phase difference between the excitation term and original signal, as shown in Figure 8.

Notably, the second harmonic manifests as a modulated wave, with its energy distributed across a specific bandwidth. According to ref. [23], the Duffing equation exhibits sensitivity solely to frequency falling within the range matching its driving frequency. Consequently, by retaining only the components that matched the driving frequency of the equation and performing trigonometric operations, Equation (9) can be simplified as Equation (10):

(10) $$\ddot{x}+0.5\dot{x}-x+x^{3}=\left(F_{\mathrm{th}}+A\cos\left(\varphi \right)\right)\sin\left(t\right)$$

Evidently, the input of the target signal increases the driving amplitude, thereby inducing an abrupt transition of the Duffing system from a critical chaotic state to a periodic state. At this point, the driving amplitude is gradually reduced until the system reverts to the critical chaotic state again, where the driving amplitude is recorded as $F_{c}$. As the system state depends solely on the driving amplitude, the driving amplitudes at the two critical chaotic states are identical, i.e., $F_{\mathrm{th}}=F_{c}+A\cos\left(\varphi \right)$, yielding Equation (11):

(11) $$F_{\mathrm{th}}-F_{c}=A\cos\left(\varphi \right)$$

In Equation (11), $F_{\mathrm{th}}$ and $F_{c}$ are known parameters; thus, the amplitude A can be determined, assuming φ=0. However, since the initial phase φ of the target signal is unknown, this study addresses the problem by implementing a phase search across the interval $\left[0,\;2\pi \right]$ by the delay traversal method. This approach effectively transforms the unknown phase problem into a peak detection problem.

The core idea of phase traversal compensation is to delay the sampling of the original signal until it is in phase with the Duffing equation, which eliminates the phase difference and facilitates amplitude identification. Specifically, for a target signal with a period T, delayed sampling is performed $M=T/t_{s}$ times within the interval $\left[0,\;T\right]$ using a step size of $t_{s}$. For the i-th delay sampling, where the delay time is $t_{d}=i\cdot t_{s}$ $\left(\mathrm{where}\;i=(0,1,\cdots M-1)\right)$ and the corresponding delayed phase is $\Delta \varphi =t_{d}$, the deviation of the system’s critical driving force satisfies Equation (12):

(12) $$F_{\mathrm{th}}-F_{c}=A\cos\left(\varphi -t_{d}\right)$$

**Figure 9:**
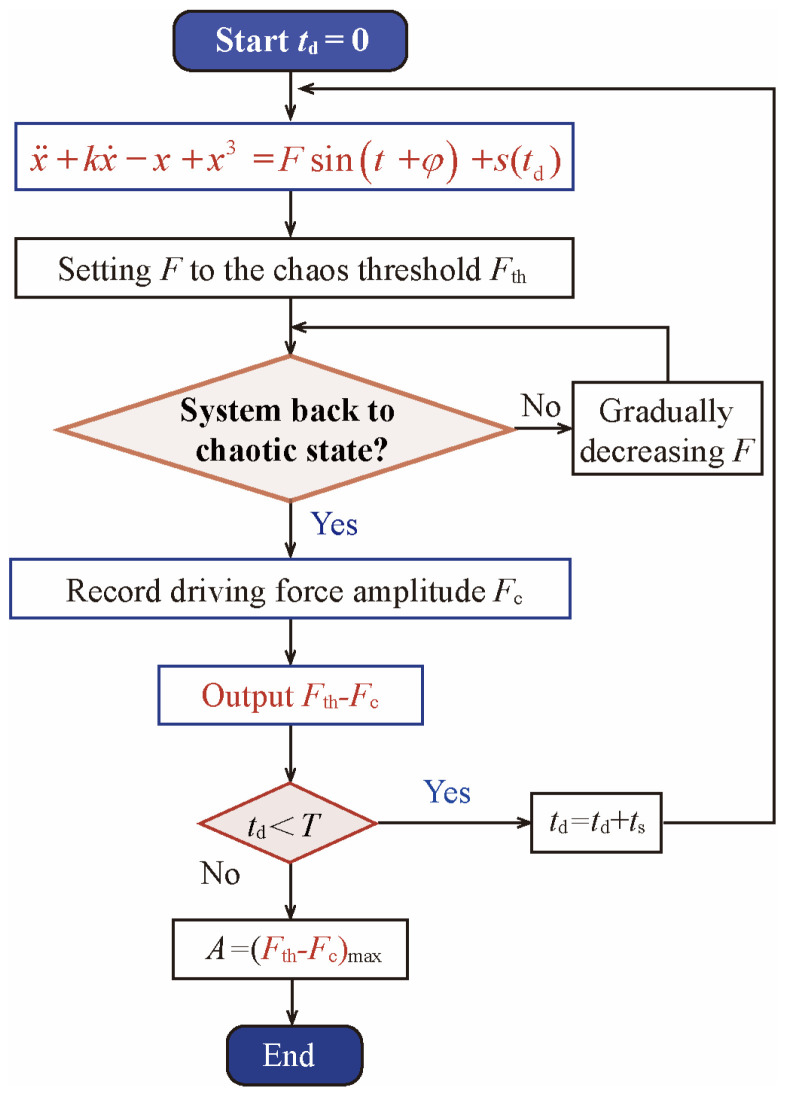
Flowchart of the time-domain signal amplitude identification based on a chaotic system.

Evidently, $\left(F_{\mathrm{th}}-F_{c}\right)$ and $t_{d}$ exhibit a cosine functional relationship. When the delayed-sampled signal is in phase with the Duffing equation, the phase difference is eliminated, as shown in Figure 8. At this point, $t_{d}=\varphi$ and ${\left(F_{\mathrm{th}}-F_{c}\right)}_{\max}=A$. Consequently, by traversing the complete phase cycle, the maximum value obtained corresponds to the amplitude A. The detailed identification procedure is illustrated in Figure 9.

### 3.3. Weak Signal Detection and Noise Immunity Analysis

**Figure 10:**
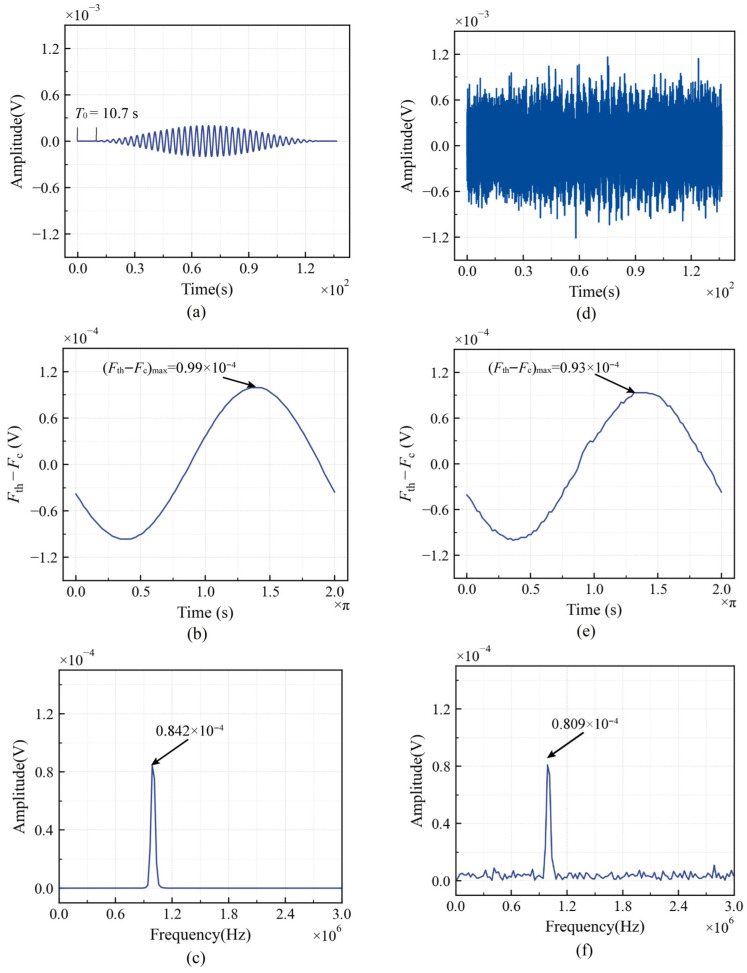
Comparison of second harmonic amplitude detection under noise-free and −10 dB conditions: (**a**) pure second harmonic signal; (**b**) the proposed method recognition result for pure second harmonic signal; (**c**) FFT recognition result for pure second harmonic signal; (**d**) −10 dB second harmonic signal; (**e**) the proposed method recognition result for pure −10 dB second harmonic signal; (**f**) FFT recognition result for pure −10 dB second harmonic signal.

The Duffing equation shown in Equation (7) is used as the detection system, with parameters set to *k* = 0.5 and *F* = 0.826808. To verify the detection system’s ability to extract second-harmonic amplitudes, a simulated signal was generated based on Equation (2), as shown in Figure 10a. The signal amplitude is *A* = 0.0001 V, the number of cycles is *n* = 20, and the center frequency is f= 1MHz. Considering the detection distance and damage causing a phase shift in the second harmonic relative to the fundamental wave, a delay input time has been set to $T_{0}=10.7\;s$, which indicates that the second harmonic is input into the system after $T_{0}$ time. To match the signal frequency with the detection system frequency, a time-scale transformation is applied to set the angular frequency of the test signal to 1.

Delayed sampling detection is performed on the signal using the method illustrated in Figure 9. By varying Δφ from 0 to 2π, the corresponding $\left(F_{\mathrm{th}}-F_{c}\right)$ for each delayed sampled signal is calculated, and the $(F_{\mathrm{th}}-F_{c})–t_{d}$ curve is plotted, as shown in Figure 10b. It can be observed that as $t_{d}$ varies, $\left(F_{\mathrm{th}}-F_{c}\right)$ initially increases and subsequently decreases. The overall trend follows a cosine function, which is in agreement with theoretical expectations. The amplitude of the test signal is determined to be $A={\left(F_{\mathrm{th}}-F_{c}\right)}_{\max}=0.99\times 10^{-4}$. The relative error of amplitude recognition is −1%, indicating that the proposed method has high recognition accuracy for a weak second harmonic. To highlight the advantages of the proposed method compared to FFT, the signal in Figure 10a was processed using a fast Fourier transform, as shown in Figure 10c. The amplitude recognition result is $0.824\times 10^{-4}$, with a relative error of −17.6%, markedly exceeding the error of the proposed method. This phenomenon occurs mainly because FFT averages the signal’s amplitude across the time domain, while the second harmonic is the time domain’s non-stationary signal.

Considering that noise from the environment and equipment is inevitable during actual testing, it is necessary to verify the proposed method’s ability to recognize signals with a low SNR. White Gaussian noise with a mean of 0 was superimposed on the signal shown in Figure 10a, where the noise power was set to 10 times that of the signal. Consequently, a weak harmonic signal with an SNR of −10 dB was generated, as shown in Figure 10d. The recognition results of the proposed method are shown in Figure 10e. It can be seen that the noise interference merely caused perturbations to the curve $(F_{\mathrm{th}}-F_{c})–t_{d}$ without altering its functional relationship, and the overall trend still exhibits a cosine variation. The amplitude of the −10 dB test signal can be obtained as $A={\left(F_{\mathrm{th}}-F_{c}\right)}_{\max}=0.93\times 10^{-4}$, and the relative error of amplitude recognition is −7%. The signal processing result of FFT is shown in Figure 10f. The amplitude recognition result is $0.809\times 10^{-4}$, with a relative error of −19.1%, which is significantly higher than that of the proposed method.

**Figure 11:**
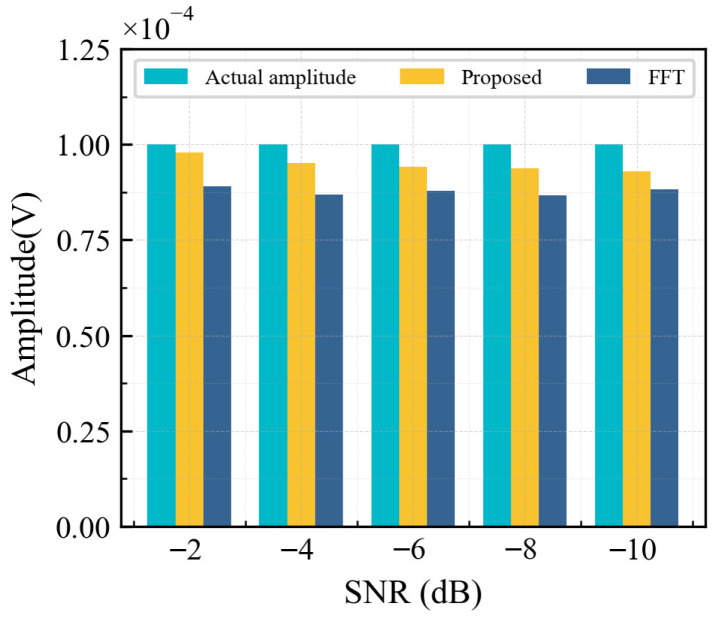
Comparison of the identification results of the proposed method with FFT.

To further illustrate the advantages of the proposed method, Figure 11 compares the amplitude recognition accuracy between our method and FFT across various SNRs. With a decreasing SNR, the errors for the proposed method and FFT gradually increase. While the FFT recognition results remain relatively stable with a slowly changing error rate, their relative errors consistently remain worse than −11%. Conversely, the proposed method maintains relative errors of less than −7%, demonstrating its superiority over FFT. In conclusion, the proposed method enables accurate, direct extraction of the second harmonic amplitude from the time domain, leading to a more precise nonlinear coefficient and improved nonlinear ultrasonic testing accuracy.

## 4. Fatigue Crack Detection Based on Time-Domain Amplitude Analysis

**Figure 12:**
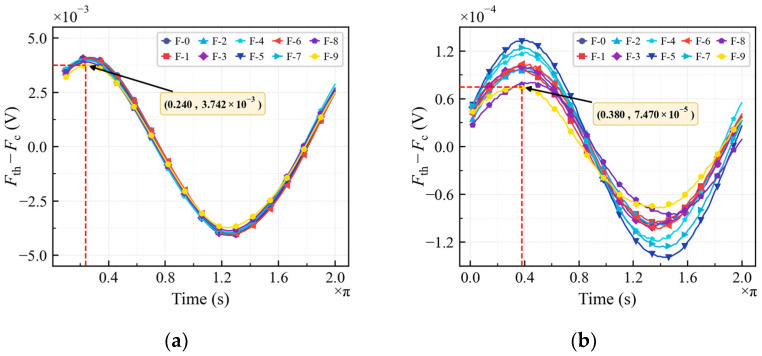
Identification results: (**a**) fundamental wave amplitude identification and (**b**) second harmonic amplitude identification.

This section applies the nonlinear ultrasonic time-domain identification based on chaos sensitivity to analyze the nonlinear ultrasonic testing results presented in Section 2.2 and determines the nonlinear coefficient. The signals were analyzed directly in the time domain without spectral analysis. The identification process consisted of two main steps: first, the target signal was extracted to identify the fundamental wave amplitude, following the analysis workflow shown in Figure 9. Subsequently, the test signal was processed using a second-order digital Butterworth notch filter with a center frequency of 0.5 MHz. This filtering was implemented directly on the discrete data via time-domain convolution. The isolated second harmonic was then identified following the same procedure. It should be noted that while the filter targets specific frequency components, the entire process—from signal preprocessing to the chaos-based amplitude identification—was executed exclusively using time-series data, without relying on FFT or spectral analysis. As shown in Figure 12a,b, both the fundamental wave and second harmonic exhibit a distinct cosine trend with respect to $\left(F_{\mathrm{th}}-F_{c}\right)$ and $t_{d}$, consistent with the theoretical results of Equation (12). Regarding the fundamental wave, owing to its high signal-to-noise ratio, the amplitude variation is minimal, and the curves from stages F-0 to F-5 nearly overlap, as shown in Figure 12a. This suggests that in the fatigue crack extension stage, damage primarily manifests as fatigue cracks that have a negligible effect on the fundamental wave amplitude; consequently, detecting early-stage fatigue damage based on fundamental wave variations proves challenging. Conversely, a slight decline in the fundamental wave amplitude is observed in operating conditions F-6 to F-9. This reduction is attributed to the transition of damage into the macroscopic crack propagation stage, where macroscopic cracks significantly impede acoustic wave propagation. Figure 12b presents the identification results of the second harmonics under corresponding conditions, demonstrating its superior sensitivity to the degree of fatigue damage. This phenomenon results from the interaction between acoustic waves and the gradually propagating fatigue cracks within the structure, as detailed in Section 3.1. Consequently, the evolution of fatigue cracks significantly alters the second harmonic amplitude. Additionally, it is noteworthy that the initial phases of the fundamental wave and the second harmonic differ, despite being extracted from the same signal segment. For instance, the F-9 result displays a fundamental wave initial phase of 0.240π and a second harmonic phase of 0.380π. This phase difference is consistent with the theory that damage induces a phase shift in the second harmonic signal relative to the fundamental wave.

**Figure 13:**
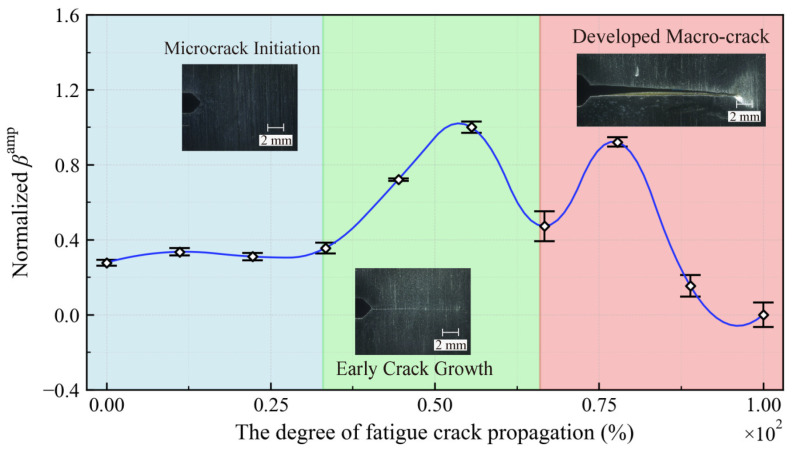
Variation in $\beta^{\mathrm{amp}}$ with the fatigue damage degree.

Table 2 lists the average amplitudes of the fundamental wave and second harmonic in all cases. Based on these data, the corresponding nonlinear coefficients $\beta^{\mathrm{amp}}=u^{2\omega }/u^{\omega }$ are calculated. The variation of $\beta^{\mathrm{amp}}$ as a function of the degree of fatigue damage is illustrated in Figure 13. As observed, the nonlinear coefficient for U71Mn steel does not increase monotonically during the fatigue process; instead, it exhibits a distinct “double-peak” characteristic associated with crack propagation. Specifically, the first peak emerges at the intermediate fatigue stage, followed by a slight decline. Subsequently, the coefficient rises to a second peak in the late fatigue stage, finally decreasing rapidly. This behavior is consistent with the findings reported by Ogi et al. [33] for carbon steel and Jaya Rao et al. [34] for the aluminum alloy AA7175-T7351. The observed double-peak characteristic suggests a complex damage evolution process during crack propagation, possibly attributed to the formation of multiple branch cracks observed in U71Mn steel [35].

As the degree of fatigue damage increases from 0% to approximately 50%, the nonlinear coefficient exhibits a gradual rise, which corresponds to the transition from micro-damage accumulation to stable crack initiation as captured in the optical images. The nonlinearity originates from the periodic contact and separation of the fatigue crack faces; consequently, as the crack length increases and the contact area expands, the nonlinear coefficient exhibits an upward trend. Previous studies have demonstrated that this nonlinearity is induced by fatigue cracks. The finding has been verified through experimental observations [36]. The first peak occurs when the degree of fatigue damage reaches approximately 50%, at which point the internal damage transitions from microcrack initiation to an early macroscopic tight crack. Subsequently, the nonlinear response decreases slightly but remains relatively high; the secondary rise observed in this process is possibly attributed to the formation of branched cracks. A second peak emerges when the degree of fatigue damage reaches approximately 80%, at which time the fatigue damage has evolved into a developed macro-crack. At this stage, the physical gap of the crack exceeds the vibration amplitude induced by the probing wave, making it insufficient to induce vibration at the macroscopic interface. This leads to a decrease in interface stress and a reduction in higher-order harmonic amplitudes. Consequently, the nonlinear coefficient drops sharply when the degree of fatigue damage exceeds 80%. Furthermore, the error bars in the Figure 13 represent the normalized standard errors of the mean of the data from the five experiments. This error analysis indicates that the detection approach remains relatively stable when the degree of fatigue damage is low (0% to approximately 50%). However, when the degree of fatigue damage exceeds 60%, the detection error increases significantly. Evidently, the nonlinear detection method is particularly effective for early-stage fatigue detection. In engineering applications, the emergence of the first peak indicates the early stage of fatigue damage, corresponding to the transition from microcrack accumulation to an initial crack propagation. Compared with linear ultrasonic techniques that only respond to macrocracks, this characteristic enables earlier damage identification and effectively advances the detection window, providing sufficient time for structural health monitoring and maintenance. The second peak corresponds to the formation and rapid propagation of macroscopic cracks, indicating that the structure has entered a critical warning state. In this case, timely reinforcement and maintenance are required to avoid sudden structural failure.

Notably, the damage mechanisms of different materials vary substantially, leading to distinct evolutionary patterns in their ultrasonic nonlinear coefficients during fatigue progression. For instance, in aluminum alloys [37], the nonlinear coefficient initially remains relatively stable before increasing significantly. By contrast, nickel-based superalloys subjected to low-cycle fatigue [38] exhibit a continuous rising trend, whereas 316 L stainless steel [39] displays a characteristic two-stage evolution consisting of a rapid initial increase followed by gradual saturation. Moreover, internal damage mechanisms differ significantly under varying loading conditions, resulting in divergent nonlinear trends. For example, although nickel-based superalloys exhibit an increasing nonlinear coefficient under low-cycle fatigue [38], they show a much slower increasing trend in acoustic nonlinearity under high-cycle fatigue. Consequently, when using nonlinear ultrasonics for structural fatigue damage assessment, a comprehensive understanding of the material-specific fatigue crack evolution behavior is essential. This topic will constitute a primary focus of our future work.

While the “double-peak” characteristic observed in this study offers a new perspective for stage-based warning, the underlying micro-mechanisms still require further quantitative clarification. Future research will focus on establishing a quantitative mapping between micro-morphological evolution and nonlinear acoustic responses. This will further refine the full-lifecycle evolution and monitoring theory for fatigue cracks.

## 5. Conclusions

To address the problem of insufficient sensitivity in existing methods for detecting fatigue damage in U71Mn rail steel, this study proposed a nonlinear ultrasonic detection method that integrates chaotic-based weak signal recognition. A probe pressure monitoring device was designed to eliminate contact nonlinearity caused by probe coupling inconsistencies. A nonlinear ultrasonic time-domain identification method based on chaotic sensitivity was proposed, and the amplitudes of the fundamental and second harmonics were quantitatively extracted. Based on this, the ultrasonic nonlinear evolutionary behavior of U71Mn rail steel during the fatigue damage process was investigated, which provided a theoretical foundation and methods for its early fatigue damage detection. The main research conclusions were summarized as follows:

(1) A nonlinear ultrasonic time-domain identification based on chaos sensitivity was developed. Challenges regarding the signal–system frequency matching and the unknown initial phase of the test signal in the Duffing detection system were resolved by time-scale transformation and phase traversal compensation. This provides an effective time-domain identification method for nonlinear ultrasonics at arbitrary frequencies.

(2) A probe pressure monitoring device was designed to eliminate contact nonlinearity caused by probe coupling inconsistencies. As the probe pressure increases, the nonlinearity coefficient transitions from a phase of rapid change to a steady state, indicating that the contact nonlinearity gradually diminishes. The stable-stage value of 16 N was chosen as the optimal pressure, under which contact nonlinearity is weak and can be ignored. This improves the robustness of nonlinear ultrasonic testing.

(3) The proposed chaos sensitivity-based nonlinear ultrasonic identification method demonstrated stronger parameter sensitivity and noise robustness than traditional FFT. When applied to a −10 dB second harmonic signal, the proposed method achieved an amplitude identification error of −7%, outperforming FFT (−11.7%). This indicated that the proposed method effectively enhances nonlinear ultrasonic amplitude identification accuracy in low SNR environments.

(4) The analysis of U71Mn rail base material demonstrated a “double-peak” characteristic in the nonlinear coefficient as fatigue damage evolved. Specifically, the first peak appeared at approximately 50% fatigue damage (corresponding to the transition from microcrack initiation to early crack growth), while the second peak emerged at approximately 80% (corresponding to the transition into a developed macro-crack). This characteristic can serve as a critical criterion for classifying rail fatigue damage stages. Additionally, the method demonstrated excellent stability during the early fatigue stage (0–50%), confirming its applicability for early damage identification. It should be noted that current research focuses on theoretical and methodological studies of nonlinear ultrasonic testing of the intrinsic material properties of U71Mn rail steel. Future research will focus on applying this theoretically viable approach to in situ inspections on operational rail lines represents.

## Figures and Tables

**Table 1:** Testing cases.

Case No.	F-0	F-1	F-2	F-3	F-4	F-5	F-6	F-7	F-8	F-9
Number of Cycles	0	15,400	30,800	46,200	61,600	77,000	92,400	107,800	123,200	138,600
$\mathrm{Degree}\;\mathrm{of}\;\mathrm{fatigue}\;\mathrm{damage}\;Fd_{a}$ (%)	0	11.11	22.22	33.33	44.44	55.55	66.67	77.78	88.89	100

**Table 2:** Nonlinear coefficients for closed cracks in U71Mn steels.

Case No.	Degree of Fatigue Damage $Fd_{a}$ (%)	$u^{2\omega }$ × 10^−4^ (V)	$u^{\omega }$ × 10^−3^ (V)	$\beta^{\mathrm{amp}}$
F-0	0%	0.960	4.086	5.751
F-1	11.11%	0.973	4.059	5.944
F-2	22.22%	0.976	4.080	5.860
F-3	33.33%	1.002	4.083	6.011
F-4	44.44%	1.175	4.032	7.231
F-5	55.56%	1.329	4.035	8.162
F-6	66.67%	1.039	4.031	6.397
F-7	77.78%	1.241	3.964	7.898
F-8	88.89%	0.800	3.875	5.334
F-9	100%	0.747	3.742	4.819

## Data Availability

Data will be available upon reasonable request.
